# Editorial: Cause or effect: role of inflammation in metabolic disorder

**DOI:** 10.3389/fendo.2024.1359605

**Published:** 2024-01-26

**Authors:** Suresh Singh Yadav, Rohini R. Nair, Kanahiya Singh

**Affiliations:** ^1^ Department of Molecular Biology and Biochemistry, Guru Nanak Dev University, Amritsar, India; ^2^ Department of Medical Biotechnology Gujarat Biotechnology University, Gandhinagar, India; ^3^ McGowan Institute for Regenerative Medicine, Department of Surgery, University of Pittsburgh, School of Medicine, Pittsburgh, PA, United States

**Keywords:** metabolic disorder, GPCR (G protein coupled receptor), fasting, endothelial (dys) function, epigenetics (DNA methylation), inflammation, intravital 2-photon microscopy, sepsis

A living organism’s ultimate need is energy, which is obtained by the metabolism of food. These metabolic processes have highly regulated multiple steps. Disruptions of these processes at single or multiple steps are collectively known as metabolic disorders. Inflammation is the response of the immune system to infections, tissue damage, or cytokine hypersecretion. It leads to the accumulation of specialized immune cells called inflammatory cells. Inflammation and tissue microenvironment play a significant role in the development of metabolic syndrome. Metabolic syndrome, such as obesity, insulin resistance, and diabetes, is associated with proinflammatory states. Low-grade inflammation is considered as the diagnostic parameter associated with metabolic syndrome. Chronic inflammation because of overnutrition, physical inactivity, and ageing results in cytokine hypersecretion, leading to metabolic disorders in predisposed individual. Different players of inflammation, such as immune cells and mediators of the innate immune system, are major contributors to the pathogenesis of metabolic disorders (1). Hence, considering inflammation in metabolic disorder as cause or effect is a matter of debate.

In this Research Topic, we aim to encourage the authors to investigate how inflammation orchestrates the onsets of metabolic disorders or *vice versa* and suggest possible therapeutic options. The research articles and reviews accepted on this topic enlist and explain important concepts and underlying mechanisms to develop a better understanding in this direction. The article by Li et al. has discussed neonatal and adult sepsis as a major impact on inflammation-related diseases. The authors used Mendelian randomization (2) methods on the data collected from public genome-wide association studies to analyze the genetic association of neonatal/adult sepsis with asthma, allergy, rheumatoid arthritis, body mass index/obesity, type 1/type 2 diabetes, and intelligence/dementia. They identified that both neonatal and adult sepsis were related to decreased body mass index and decreased risk of obesity, respectively. This study calls for the urgent need for a similar study to determine the significant detrimental effect of sepsis on a wide range of physiological systems.

In a review article, Bhamidipati et al. explained how tissue epigenome is affected by persistent hyperglycemia in T2DM subjects which, in turn, causes diabetic vasculopathy. Through elaborating the differential abundance of epigenetic modifiers due to hyperglycemia (3, 4), they categorically defined why diabetes is not only a genetic disease but also a disease that relies on environmental cues for expression. They categorically presented the examples of how epigenetic regulators such as DNA methylation and histone modification can affect the expression of microRNAs during pathogenic angiogenesis in diabetics (5). This article also advocated the use of novel intravital microscopy tools in diabetic wound healing research to reveal the high-resolution composition of the newly formed granulation tissue and how vascular tissue elements reorganize post-injury (**Figure 1**). The evidence presented in this article strongly justifies the need to develop epigenetics-based therapies to reactivate the pathways silenced in chronic wounds (6).

**Figure 1:**
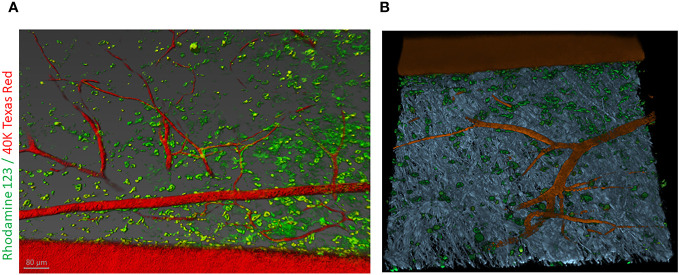
Intravital labeling for two-photon microscopic imaging of adult mouse skin vasculature. **(A)** Texas Red 40 kDa dextran marked the perfused vasculature and Rhodamine-123 marked the cytoplasm of cells present nearby the vasculature containing the mitochondria. Scale bar = 80 µm. **(B)** Using Imaris software, the three-dimensional vessel structure was reconstructed from a region of interest from **(A)**. The gray color represents second-harmonic generation indicative of collagen in the skin tissue. Figure adapted from Bhamidipati et al..

In an interesting research article, Hao et al. investigated the effect of a 14-day fasting regimen on the balance between skeletal muscle and adipose tissue composition in eighty healthy human subjects with a goal to evaluate the association with inflammatory factors. Their results demonstrated that a combination of appropriate prebiotic and mineral supplements protects individuals from gut injury or physical discomfort during this fasting regimen.

In continuation with the research theme, Liu et al. discussed the features of different mechanotransduction pathways and how understanding their cellular ontogeny of adipose tissue is important to underline the development of adipocytes involved in cardiovascular disorders. This knowledge is important as a disproportionate distribution of adipose tissue may induce systemic lipotoxicity and insulin resistance during the development of cardiovascular disease. They went on to explain the idea of how therapeutic interventions targeting adipose tissue can improve cardiovascular health in patients with obesity and diabetes. Logically explaining the mechanisms behind the involvement of adipose tissue in inflammation, Duncan et al. associated metabolite sensing G protein-coupled receptors (GPCRs) with organ-wide chronic low-grade inflammation observed in metabolic disorders. They systematically discussed how three adipocyte-associated metabolic intermediates—(i) long-chain fatty acids, (ii) hydroxy carboxylic acids, and (iii) succinate—may signal through GPCRs expressed on different cell types such as resident adipocytes and on invading macrophages. This signaling, in turn, controls the interaction between adipocyte and macrophages and regulates inflammation in immune cells and lipolytic pathways in adipose tissue. In conclusion, they explained the need of proper understanding of metabolic–immune interactions to establish how targeting GPCRs opens a new avenue in the management and treatment of metabolic disorders.

## Author contributions

SY: Writing – original draft, Writing – review & editing. RN: Writing – original draft, Writing – review & editing. KS: Writing – original draft, Writing – review & editing.
